# Supplementary material for: Unraveling the Tropaeolum majus L. (Nasturtium) Root-Associated Bacterial Community in Search of Potential Biofertilizers
Source: Microorganisms. 2022 Mar 17;10(3):638. doi: 10.3390/microorganisms10030638 (PMC8950702; doi:10.3390/microorganisms10030638)
Supplement: Supplementary file 1 [file microorganisms-10-00638-s001.zip › microorganisms-1632563-supplementary.pdf]

microorganisms

MDPI

Unraveling the *Tropaeolum majus* L. (nasturtium) Root-Associated Bacterial Community in Search of Potential Biofertilizers

Isabella Dal'Rio, Jackeline Rossetti Mateus and Lucy Seldin *

Supplementary Material

Table S1. Physicochemical properties of the rhizosphere (n = 5) and bulk soil (n = 5) associated with *Tropaeolum majus*.

Parameters	Rhizosphere	Bulk soil
pH	5.6	5.5
Organic Matter (mg/dm³)	350	410
Total Organic Carbon (mg/dm³)	200	240
C:N ratio	8.16	6.03
Cation exchange capacity (cmolc/dm³)	12.21	13.07
Sum-of-bases (cmolc/dm³)	10.31	10.87
V%	83	84
H° (cmolc/dm³)	1.9	2.2
Al^{3+} (cmolc/dm³)	0	0
m%	0	0
**Macronutrients**		
P (mg/dm³)	159	160
Ca (cmolc/dm³)	7.2	8.2
Mg (cmolc/dm³)	2.3	2.3
K (cmolc/dm³)	0.6	0.19
S (mg/dm³)	36	6
N (ppm)	2449.5	3980.43
**Micronutrients**		
B (mg/dm³)	1.42	1.69
Cu (mg/dm³)	3.9	3.3
Fe (mg/dm³)	29	23
Mn (mg/dm³)	42	45.2
Zn (mg/dm³)	23	22
Co (mg/dm³)	0.3	0.4
Mo (mg/dm³)	0.2	0.1

Table S2. Plant growth-promoting ability of the 236 bacterial isolates to phosphate mineralization (PM), phosphate solubilization (PS), siderophore production (SID), production of antimicrobial substances (AMS), production indole-related compounds (IRCs) and presence of *nifH* gene (*nifH*).

Strain	PM	PS	SID	AMS	IRCs	*nifH*
**Plant 1**						
E1	+	+	+	+	+	+
E2	+	−	+	−	+	−
E3	−	−	−	−	−	−
E4	+	+	+	+	+	+
E5	+	−	+	−	+	−
E6	+	+	+	+	+	+

E7	+	+	+	+	−	+
E8	+	+	+	+	+	−
E9	+	−	+	−	+	−
E10	+	−	+	−	+	−
E11	+	+	+	+	+	−
E12	+	−	+	+	+	−
E13	+	−	+	+	−	−
E14	+	+	+	+	+	+
E15	+	+	+	+	+	−
E16	+	−	+	+	+	−
E17	+	+	+	+	+	−
E18	+	+	+	+	+	−
E19	+	−	−	−	−	−
E20	+	+	−	+	−	−
E21	+	+	+	+	−	−
E22	+	−	+	+	−	−
E23	+	+	+	+	−	−
E24	+	+	+	+	+	+
E25	−	+	+	−	−	−
E26	+	+	+	+	+	+
E27	+	+	+	+	+	+
E28	+	+	+	+	+	+
E29	+	+	+	+	+	−
E30	+	+	+	+	−	−
E31	−	+	+	+	−	−
E32	−	−	+	+	+	−
E33	+	+	+	−	+	−
E34	+	+	+	+	+	+
E35	+	+	+	+	+	−
E36	+	−	+	−	+	−
E37	+	−	+	+	−	−
E38	+	+	+	+	−	−
E39	+	−	+	+	−	−
E40	+	+	+	+	−	−
E41	+	−	+	+	−	−
E42	+	−	+	+	−	−
E43	−	−	+	−	−	+
E44	+	+	+	+	−	−
E45	+	+	+	+	−	−
E46	−	+	+	−	−	+
E47	−	+	+	+	−	−
E48	−	−	+	+	−	−
E49	+	+	+	+	−	−
**Plant 2**						
E50	+	+	+	+	+	+
E51	+	+	+	−	−	−
E52	+	+	+	+	+	+
E53	+	+	+	+	+	+
E54	+	+	+	+	+	+
E55	+	+	+	−	+	+
E56	+	−	+	−	+	+

E57	+	+	+	+	+	−
E58	+	+	−	−	−	−
E59	+	+	+	−	−	−
E60	+	+	+	+	+	+
E61	+	−	+	+	+	−
E62	+	+	+	−	+	+
E63	+	+	+	+	−	−
E64	+	+	+	+	+	−
E65	+	−	+	−	−	−
E66	+	+	+	+	−	−
E67	+	+	+	+	+	−
E68	+	+	+	+	−	−
E69	+	+	+	+	−	−
E70	+	−	+	−	+	−
E71	+	−	+	−	−	−
E72	+	+	+	+	−	−
E73	+	+	+	−	−	−
E74	+	−	+	+	+	−
E75	+	+	+	+	+	−
E76	+	−	+	−	−	−
E77	+	+	+	+	+	−
E78	+	+	+	+	+	−
E79	+	+	+	+	+	+
E80	+	+	+	+	+	−
E81	+	+	+	+	−	−
E82	+	−	+	+	−	−
E83	−	+	+	−	+	−
E84	+	+	+	−	−	−
E85	+	−	−	+	−	−
E86	+	−	−	+	+	−
E87	+	−	−	+	−	−
E88	+	+	+	−	+	−
E89	+	−	−	+	+	−
E90	+	−	+	+	+	−
E91	+	−	+	+	+	−
E92	+	+	+	+	+	−
E93	−	+	+	−	−	−
E94	−	+	−	+	−	−
E95	+	+	+	+	+	−
E96	−	+	+	−	−	−
E97	+	−	+	+	+	−
E98	+	+	−	+	−	−
E99	+	+	+	−	−	−
E100	+	−	−	−	−	−
E101	+	+	+	+	+	−
E102	+	−	+	+	+	−
E103	+	+	+	+	+	−
E104	+	−	−	+	+	−
E106	−	+	+	−	−	−
E107	−	−	+	−	−	−
E108	+	+	+	−	+	−

E109	−	−	−	−	−	−
E110	−	−	−	−	−	−
E111	+	−	−	−	−	−
E112	+	−	−	+	+	−
E113	+	−	−	+	−	−
E114	+	+	+	−	+	−
**Plant 3**						
E115	−	−	−	−	−	−
E116	+	+	+	−	−	−
E117	−	+	−	+	−	−
E118	−	+	−	+	−	−
E119	−	+	−	+	−	−
E120	+	+	+	+	−	−
E121	−	+	−	+	−	−
E122	+	+	+	+	−	−
E123	+	+	−	+	−	−
E124	−	−	−	−	−	−
E125	−	+	−	+	−	−
E126	+	+	+	+	−	−
E127	−	+	−	+	−	−
E128	−	+	−	+	−	−
E129	−	+	−	−	−	−
E130	−	+	−	+	−	−
E131	−	+	−	+	−	−
E132	−	+	−	+	−	−
E133	+	+	+	+	−	+
E134	+	−	−	+	−	−
E135	−	−	−	−	−	−
E136	−	+	−	+	−	−
E137	−	+	−	−	−	−
E138	−	+	−	−	−	−
**Plant 4**						
E140	−	−	−	+	−	−
E141	+	+	+	−	+	−
E142	+	−	−	−	−	−
E143	+	+	+	−	+	+
E144	+	+	+	−	+	−
E145	−	−	−	−	−	−
E146	−	−	−	−	−	−
E147	+	+	+	−	+	−
E148	+	+	+	−	+	−
E149	−	−	−	+	−	−
E150	+	+	−	+	−	−
E151	−	−	−	−	−	−
E152	+	+	+	+	+	+
E153	−	+	−	+	+	−
E154	+	+	−	+	−	−
E155	−	+	−	+	−	−
E156	+	+	−	+	−	−
E157	−	−	−	−	−	−
E158	+	+	+	+	+	−

E159	−	−	−	−	−	−
E160	+	+	+	−	+	−
E161	−	−	−	+	−	−
E162	+	+	+	+	+	+
E163	−	−	−	+	+	−
E164	−	+	−	−	−	−
E165	−	+	−	−	−	−
E166	−	+	−	+	+	−
E167	−	−	+	+	+	−
E168	+	+	+	−	+	−
E169	−	+	−	−	−	−
E170	+	−	−	−	−	−
E171	−	+	+	−	+	−
E172	−	+	+	−	+	−
E173	−	+	−	−	−	−
E174	+	+	−	−	−	+
E175	−	+	−	+	−	−
E176	+	+	−	+	−	−
**Plant 5**						
E177	−	+	+	+	+	−
E178	−	+	+	+	+	+
E179	−	+	+	+	+	−
E180	−	+	−	+	−	−
E181	−	+	+	+	+	−
E182	−	+	+	+	+	−
E183	−	+	+	+	+	−
E184	−	+	+	+	+	−
E185	+	+	+	−	−	−
E186	−	+	+	+	−	−
E187	−	+	+	+	+	−
E188	+	−	−	−	−	−
E189	−	+	+	−	−	+
E190	+	+	+	+	+	−
E191	−	+	+	+	+	−
E192	−	+	+	−	+	−
E193	+	+	+	+	+	−
E194	+	+	+	+	+	−
E195	−	+	+	+	−	−
E196	−	+	+	+	+	−
E197	+	+	+	+	+	−
E198	+	+	+	+	+	−
E199	+	+	+	+	+	−
E200	−	−	+	−	+	−
E201	+	+	+	+	−	+
E202	−	+	+	−	+	−
E203	−	+	+	+	+	−
E204	+	+	+	+	+	−
E205	−	−	+	−	+	−
E206	+	+	+	+	+	−
E207	−	+	+	+	−	−
E208	−	+	+	+	+	−

E209	−	+	+	+	+	−
E210	+	+	+	+	+	−
E211	−	+	+	−	+	−
E212	−	+	+	+	−	−
E213	−	+	+	+	−	−
E214	−	+	+	−	−	−
E215	+	−	−	+	−	−
E216	+	−	−	+	−	−
E217	+	−	−	+	−	−
E218	+	−	−	+	−	−
E219	−	−	+	−	−	−
E220	−	+	−	−	−	−
E221	+	+	+	−	−	−
E222	+	+	−	+	−	−
E223	+	+	+	−	−	−
E224	+	−	−	+	−	−
E225	+	+	−	+	−	−
E226	+	−	−	+	+	−
E227	+	+	−	+	−	−
E228	+	+	+	−	−	−
E229	+	+	−	+	−	−
E230	−	+	−	−	−	−
E231	+	−	−	+	−	−
E232	+	−	−	+	−	−
E233	+	+	−	−	−	−
E234	+	+	+	−	−	−
E235	+	+	+	−	−	−
E236	+	+	+	+	−	−
E237	−	+	−	+	+	−
E238	+	+	+	+	−	+

Table S3. Molecular identification of 76 endophytic bacteria isolated from *Tropaeolum majus* through 16S rRNA sequencing. All strains were positive in at least four out of six plant growth-promoting tests. The fragment size of each sequence is provided in base pairs (bp). The first hits in the BLASTn database are presented, and identities higher than 97% were considered to identify the strains at the genus level.

Strain	Fragment Size (bp)	Identity	Closely Related Taxa (BLASTn)	Final Identification (Genus Level)
**E1**	1489	>98%	*Raoultella terrigena* *Raoultella ornithinolytica* *Klebsiella aerogenes*	*Raoultella/Klebsiella*
**E4**	1545	>98%	*Raoultella terrigena* *Raoultella ornithinolytica* *Klebsiella aerogenes*	*Raoultella/Klebsiella*
**E6**	1544	>98%	*Raoultella terrigena* *Raoultella ornithinolytica* *Klebsiella aerogenes*	*Raoultella/Klebsiella*
**E8**	1542	>99%	*Enterobacter mori* *Enterobacter asburiae* *Enterobacter cloacae*	*Enterobacter*
**E11**	1503	>99%	*Enterobacter asburiae* *Enterobacter cloacae* *Enterobacter roggenkampii*	*Enterobacter*

**E12**	1518	>99%	*Staphylococcus warneri Staphylococcus pasteuri*	*Staphylococcus*
**E14**	1504	>98%	*Raoultella terrigena Raoultella ornithinolytica Klebsiella aerogenes*	*Raoultella/Klebsiella*
**E15**	1506	>99%	*Enterobacter asburiae Enterobacter cloacae Enterobacter roggenkampii*	*Enterobacter*
**E16**	1503	>99%	*Enterobacter asburiae Enterobacter cloacae Enterobacter roggenkampii*	*Enterobacter*
**E17**	1503	>99%	*Serratia plymuthica Serratia inhibens Serratia liquefaciens*	*Serratia*
**E23**	1510	>98%	*Enterobacter asburiae Enterobacter cloacae Enterobacter roggenkampii*	*Enterobacter*
**E26**	1055	>97%	*Raoultella terrigena Raoultella ornithinolytica Klebsiella aerogenes*	*Raoultella/Klebsiella*
**E27**	1544	>98%	*Raoultella terrigena Raoultella ornithinolytica Klebsiella aerogenes*	*Raoultella/Klebsiella*
**E29**	1543	>99%	*Enterobacter asburiae Enterobacter cloacae Enterobacter roggenkampii*	*Enterobacter*
**E33**	1544	>98%	*Serratia plymuthica Serratia inhibens Serratia liquefaciens*	*Serratia*
**E34**	1545	>99%	*Serratia proteamaculans Serratia grimesii Serratia liquefaciens*	*Serratia*
**E35**	1542	>98%	*Pantoea agglomerans Pantoea vagans Pantoea ananatis*	*Pantoea*
**E45**	1551	>99%	*Bacillus* sp.	*Bacillus*
**E49**	1520	>99%	*Bacillus* sp.	*Bacillus*
**E50**	1546	>99%	*Serratia proteamaculans Serratia grimesii Serratia liquefaciens*	*Serratia*
**E52**	1546	>99%	*Serratia proteamaculans Serratia grimesii Serratia liquefaciens*	*Serratia*
**E53**	1543	>98%	*Raoultella terrigena Raoultella ornithinolytica Klebsiella aerogenes*	*Raoultella/Klebsiella*
**E54**	1547	>99%	*Serratia proteamaculans Serratia grimesii Serratia liquefaciens*	*Serratia*
**E55**	1552	>99%	*Serratia proteamaculans Serratia grimesii Serratia liquefaciens*	*Serratia*
**E57**	1543	>99%	*Serratia plymuthica Serratia inhibens Serratia liquefaciens*	*Serratia*

**E60**	1545	>98%	*Raoultella terrigena Raoultella ornithinolytica Klebsiella aerogenes*	*Raoultella/Klebsiella*
**E62**	1549	>98%	*Raoultella terrigena Raoultella ornithinolytica Klebsiella aerogenes*	*Raoultella/Klebsiella*
**E63**	1544	>99%	*Pantoea agglomerans Pantoea vagans Pantoea ananatis*	*Pantoea*
**E64**	1546	>99%	*Serratia proteamaculans Serratia grimesii Serratia liquefaciens*	*Serratia*
**E66**	1550	>97%	*Pantoea agglomerans Pantoea vagans Pantoea ananatis*	*Pantoea*
**E67**	1545	>98%	*Serratia plymuthica Serratia inhibens Serratia liquefaciens*	*Serratia*
**E68**	1542	>98%	*Enterobacter* sp.	*Enterobacter*
**E69**	1540	>99%	*Pseudomonas fluorescens Pseudomonas kribbensis Pseudomonas chlororaphis*	*Pseudomonas*
**E72**	1068	>99%	*Serratia entomophila Serratia plymuthica Serratia marcenses*	*Serratia*
**E77**	1542	>99%	*Serratia proteamaculans Serratia grimesii Serratia liquefaciens*	*Serratia*
**E78**	1547	>99%	*Serratia proteamaculans Serratia grimesii Serratia liquefaciens*	*Serratia*
**E79**	1544	>98%	*Raoultella terrigena Raoultella ornithinolytica Klebsiella aerogenes*	*Raoultella/Klebsiella*
**E80**	1550	>98%	*Pantoea agglomerans Pantoea vagans*	*Pantoea*
**E81**	1543	>98%	*Serratia proteamaculans Serratia grimesii Serratia liquefaciens*	*Serratia*
**E88**	1545	>98%	*Pantoea agglomerans Pantoea vagans Pantoea ananatis*	*Pantoea*
**E92**	1555	>99%	*Bacillus thurigiensis Bacillus cereus*	*Bacillus*
**E95**	1558	>99%	*Bacillus thurigiensis Bacillus cereus*	*Bacillus*
**E97**	1561	>99%	*Bacillus thurigiensis Bacillus cereus*	*Bacillus*
**E101**	1504	>98%	*Klebsiella michiganensis Klebsiella oxytoca Klebsiella grimonti*	*Klebsiella*

**E102**	1506	>98%	*Klebsiella michiganensis Klebsiella oxytoca Klebsiella grimonti*	*Klebsiella*
**E108**	1012	>97%	*Mycolicibacterium phocaium Mycolicibacterium mucogenicum*	*Mycolicibacterium*
**E114**	1508	>99%	*Serratia liquefaciens Serratia proteamaculans Serratia grimesii*	*Serratia*
**E126**	1506	>99%	*Serratia inhibens Serratia plymuthica*	*Serratia*
**E133**	1512	>98%	*Raoultella terrigena Raoultella ornithinolytica Klebsiella aerogenes*	*Raoultella/Klebsiella*
**E141**	1506	>98%	*Serratia ficaria Serratia entomophila Serratia plymuthica*	*Serratia*
**E147**	1003	>97%	*Serratia ficaria Serratia entomophila Serratia plymuthica*	*Serratia*
**E148**	1504	>99%	*Enterobacter asburiae Enterobacter cloacae Enterobacter mori*	*Enterobacter*
**E152**	1506	>98%	*Raoultella terrigena Raoultella ornithinolytica Klebsiella aerogenes*	*Raoultella/Klebsiella*
**E158**	1508	>99%	*Citrobacter freundii Citrobacter portucalensis Citrobacter braakii*	*Citrobacter*
**E160**	1506	>98%	*Serratia ficaria Serratia entomophila Serratia plymuthica*	*Serratia*
**E162**	1504	>98%	*Raoultella terrigena Raoultella ornithinolytica Klebsiella aerogenes*	*Raoultella/Klebsiella*
**E177**	1511	>98%	*Serratia marcescens Serratia ureilytica Serratia surfactantfaciens*	*Serratia*
**E179**	1092	>97%	*Enterobacter asburiae Enterobacter cloacae Enterobacter mori*	*Serratia*
**E181**	1505	>99%	*Serratia marcescens Serratia ureilytica Serratia surfactantfaciens*	*Serratia*
**E182**	1507	>99%	*Serratia marcescens Serratia ureilytica*	*Serratia*
**E183**	1487	>99%	*Serratia marcescens Serratia ureilytica*	*Serratia*
**E184**	1507	>98%	*Serratia marcescens Serratia ureilytica*	*Serratia*
**E187**	1003	>97%	*Serratia proteamaculans Serratia grimesii Serratia liquefaciens*	*Serratia*

**E190**	1514	>98%	*Serratia marcescens* *Serratia ureilytica*	*Serratia*
**E193**	1504	>99%	*Serratia marcescens* *Serratia ureilytica*	*Serratia*
**E194**	1506	>99%	*Serratia marcescens* *Serratia ureilytica*	*Serratia*
**E197**	1511	>99%	*Serratia marcescens* *Serratia ureilytica* *Serratia surfactantfaciens*	*Serratia*
**E198**	1506	>99%	*Serratia marcescens* *Serratia ureilytica*	*Serratia*
**E199**	1513	>98%	*Serratia marcescens* *Serratia ureilytica* *Serratia surfactantfaciens*	*Serratia*
**E203**	1006	>97%	*Serratia marcescens* *Serratia surfactantfaciens*	*Serratia*
**E204**	1506	>99%	*Serratia marcescens* *Serratia ureilytica* *Serratia surfactantfaciens*	*Serratia*
**E206**	1508	>99%	*Serratia marcescens* *Serratia ureilytica*	*Serratia*
**E208**	1508	>99%	*Serratia marcescens* *Serratia ureilytica*	*Serratia*
**E209**	1503	>99%	*Serratia marcescens* *Serratia ureilytica*	*Serratia*
**E210**	1505	>99%	*Serratia marcescens* *Serratia ureilytica*	*Serratia*
**E238**	1562	>98%	*Paenibacillus polymyxa Paenibacillus kribbensis Paenibacillus peoriae*	*Paenibacillus*

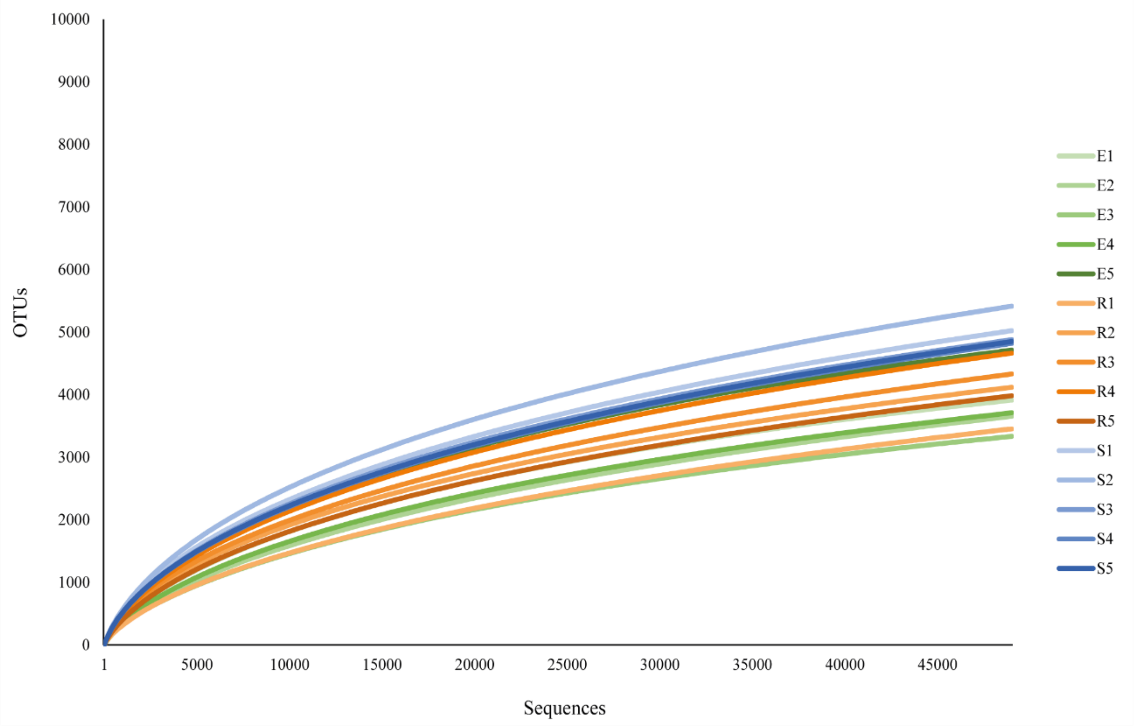

Figure S1. Rarefaction curves of the replicates of the three sites associated with *Tropaeolum majus* L.: endosphere (E–green), rhizosphere (R–orange) and bulk soil (S–blue).

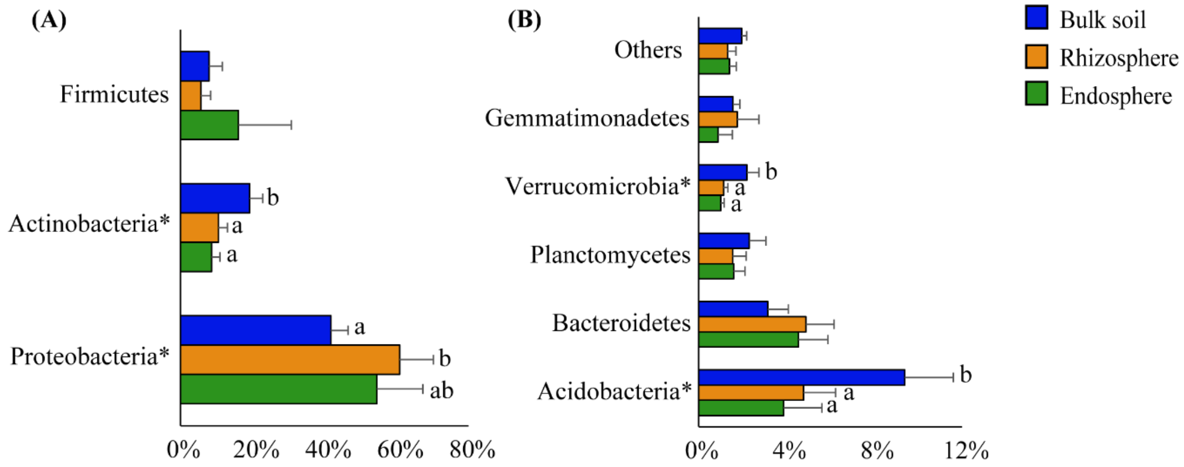

Figure S2. Relative abundance of eight bacterial phyla identified with 16S rRNA amplicon metagenomic sequencing of bulk soil, rhizosphere and endosphere samples associated with *Tropaeolum majus*. Asterisks indicate statistically significant differences among the phyla. Different letters next to the error bars refer to the sites that were significantly different within one phylum (Tukey's test, $p < 0.05$). (**A**) Up to 80% relative abundance and (**B**) up to 12% relative abundance.

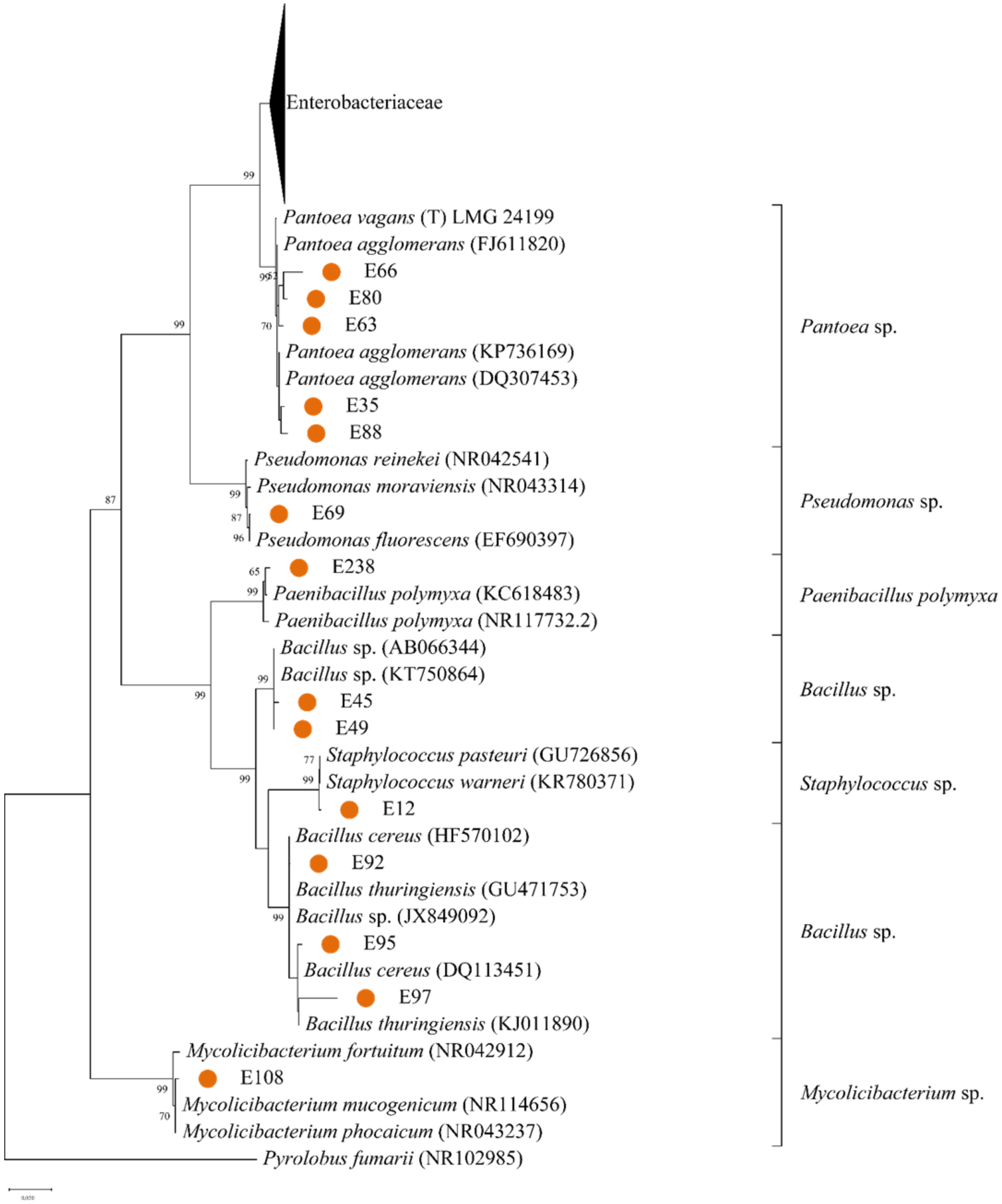

Figure S3. Maximum likelihood tree of the multiple alignment of the 16S rRNA-encoding gene of *Tropaeolum majus* isolated strains (marked with orange circles) and related species. The GenBank accession number of each sequence is shown in parentheses. The clades associated with the Enterobacteriaceae family are collapsed to a better comprehension of the tree. Bootstrap values are expressed as percentages of 500 replications and are shown at branch points. *Pyrolobus fumarii* was used as the outgroup. Bar = substitutions per nucleotide position.

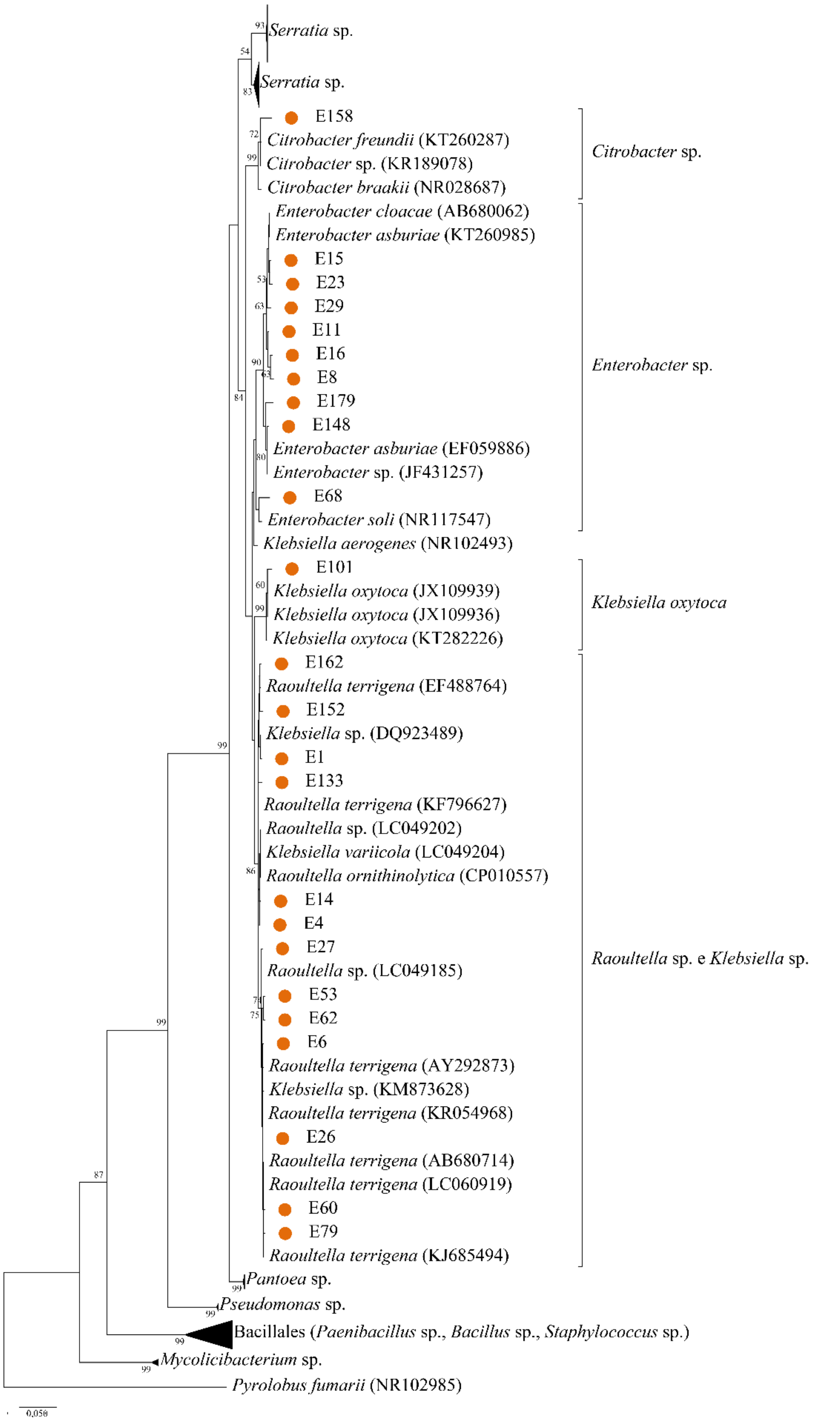

Figure S4. Maximum likelihood tree of the multiple alignment of the 16S rRNA-encoding gene of *Tropaeolum majus* isolated strains (marked with orange circles) and related species. The GenBank accession number of each sequence is shown in parentheses. The clades associated with the Enterobacteriaceae family are highlighted (with the exception of the *Serratia* genus), and the remaining clades are collapsed to a better comprehension of the tree. Bootstrap values are expressed as percentages of 500 replications and are shown at branch points. *Pyrolobus fumarii* was used as the outgroup. Bar = substitutions per nucleotide position.

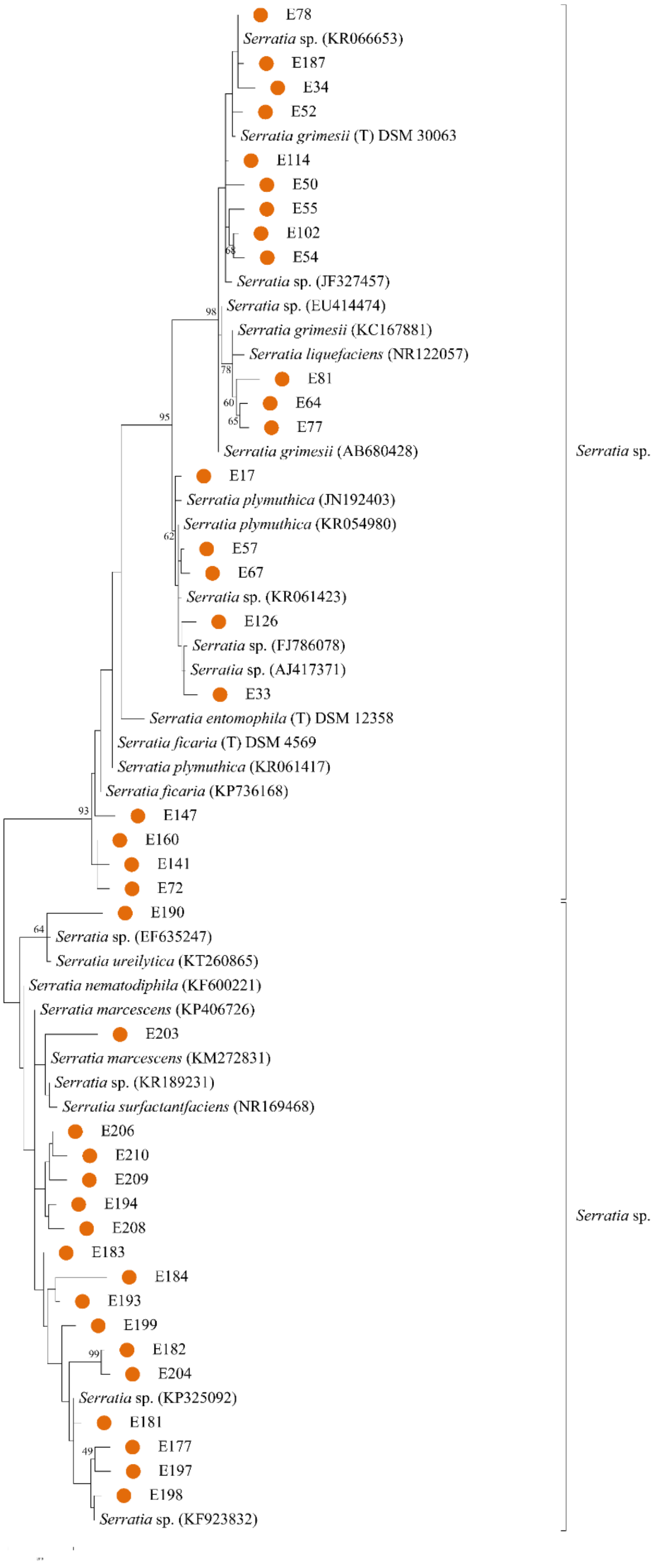

Figure S5. Maximum likelihood tree of the multiple alignment of the 16S rRNA-encoding gene of *Tropaeolum majus* isolated strains (marked with orange circles) and related species. The GenBank accession number of each sequence is shown in parentheses. The clades associated with the *Serratia* genus are highlighted, and the remaining clades are hidden to a better comprehension of the tree. Bootstrap values are expressed as percentages of 500 replications and are shown at branch points. *Pyrolobus fumarii* was used as the outgroup. Bar = substitutions per nucleotide position.
